# Clinical characteristics and genetics of ten Chinese children with PRRT2-associated neurological diseases

**DOI:** 10.3389/fped.2022.997088

**Published:** 2022-11-17

**Authors:** Meiyan Liu, Xiaoang Sun, Longlong Lin, Xiaona Luo, Simei Wang, Chunmei Wang, Yuanfeng Zhang, Quanmei Xu, Wuhen Xu, Shengnan Wu, Xiaoping Lan, Yucai Chen

**Affiliations:** ^1^ Department of Neurology, Shanghai Children’s Hospital, School of Medicine, Shanghai Jiaotong University, Shanghai, China; ^2^ Department of Clinical Laboratory, Shanghai Children’s Hospital, School of Medicine, Shanghai Jiaotong University, Shanghai, China; ^3^ National Health Commission (NHC), Key Laboratory of Medical Embryogenesis and Developmental Molecular Biology and Shanghai Key Laboratory of Embryo and Reproduction Engineering, Shanghai, China

**Keywords:** proline-rich transmembrane protein 2 (PRRT2), synaptosome-associated protein 25 (SNAP 25), expression, nervous system-related diseases, whole-exome genome sequencing

## Abstract

**Background:**

Proline-rich transmembrane protein 2 (PRRT2) plays an important role in the central nervous system and mutations in the gene are implicated in a variety of neurological disorders. This study aimed to summarize the clinical characteristics and gene expression analysis of neurological diseases related to the *PRRT2* gene and explore the clinical characteristics, therapeutic effects, and possible pathogenic mechanisms of related diseases.

**Methods:**

We enrolled 10 children with *PRRT2* mutation-related neurological diseases who visited the Children's Hospital affiliated with the Shanghai Jiaotong University School of Medicine/Shanghai Children's Hospital between May 2017 and February 2022. Video electroencephalography (VEEG), cranial imaging, treatment regimens, gene results, and gene expression were analyzed. Genetic testing involved targeted sequencing or whole-exome genome sequencing (WES). We further analyzed the expression and mutation conservation of PRRT2 and synaptosome-associated protein 25 (SNAP25) in blood samples using quantitative polymerase chain reaction (qPCR) and predicted the protein structure. Summary analysis of the reported gene maps and domains was also performed.

**Results:**

Ten children with *PRRT2* gene mutations were analyzed, and 4 mutations were identified, consisting of 2 new (c.518A > C, p.Glu173 Ala; c.879 + 112G > A, p.?) and two known (c. 649 dup, p. Arg217Profs * 8; c. 649 del, p. Arg217Glufs * 12) mutations. Among these mutations, one was *de novo*(P6), and three could not be determined because one parent refused genetic testing. The clinical phenotypes were paroxysmal kinesigenic dyskinesia (PKD), benign familial infantile epilepsy (BFIE), epilepsy, infantile spasms, and intellectual disability. The qPCR results showed that *PRRT2* gene expression levels were significantly lower in children and parent carriers than the control group. The *SNAP25* gene expression level of affected children was significantly lower (*P* ≤ 0.001) than that of the control group. The mutation sites reported in this study are highly conserved in different species. Among the various drugs used, oxcarbazepine and sodium valproate were the most effective. All 10 children had a good disease prognosis, and 8 were completely controlled with no recurrence, whereas 2 had less severe and fewer seizures.

**Conclusion:**

Mutation of *PRRT2* led to a significant decrease in its protein expression level and that of SNAP25, suggesting that the mutant protein may lead to the loss of its function and that of related proteins. This mutation site is highly conserved in most species, and there was no significant correlation between specific PRRT2 genotypes and clinical phenotypes. Asymptomatic carriers also have decreased gene expression levels, suggesting that more factors are involved.

## Introduction

Proline-rich transmembrane protein 2 (*PRRT2*) is a gene located on chromosome 16 which contains four exons, and exons 2–4 encode polynucleotides containing 340 amino acids and domain protein. The N-terminal extracellular domain of PRRT2 (amino acids 1–268) contains a proline-rich domain (PRD; amino acids 131–216), and the C-terminal domain has two transmembrane domains (amino acids 268–289 and 318–338). PRRT2 is expressed throughout the central nervous system and is highly expressed in the cerebral cortex, basal ganglia, and cerebellum (1, 2). The expression levels in other organs are low and almost negligible, which further confirms that this protein plays an important role in the central nervous system (3).

Various *PRRT2* mutations include frameshift, missense, and nonsense mutations, and most cause protein truncation or activate nonsense-mediated mRNA degradation, resulting in loss of function of the protein product (4). PRRT2 gene mutations are mostly code-shifting mutations, among which c.649dupC is a hotspot mutation, with incomplete penetrance and *de novo* mutations (5, 6). Wild-type PRRT2 is a synaptic protein localized in the presynaptic membrane and axon terminals (7) and variants may lead to mislocalization, which, in turn, affects the function of related proteins. PRRT2 interacts with synaptosome-associated protein 25 (SNAP25) at the presynaptic membrane and may play a role in synaptic vesicle fusion and neurotransmitter release (8). Mutations in the *PRRT2* gene are associated with various neurological seizure-inducing disorders, including PKD (1, 9), BFIE (2, 10), and familial infantile convulsions with paroxysmal choreoathetosis (infantile convulsions and choreoathetosis, ICCA) (3). In addition to these three most common and predominant clinical phenotypes, other childhood-onset movement disorders, various forms of seizures, headaches, sleep disturbances, and intellectual disabilities have also been described (11–14). Relative to the clinical phenotype, there are few studies on the evaluation of drug effects in PRRT2-related diseases. Ion channel blockers such as oxcarbazepine (OXC) and VPA are the first choice for the treatment of children with episodic diseases caused by PRRT2 gene mutations (15).

*PRRT2* gene mutations, protein function, and clinical phenotype exhibit considerable diversity. Consequently, summarizing the clinical phenotype and treatment effects and analyzing the possible pathogenic mechanisms of gene mutations are of significance to the diagnosis and treatment of the PRRT2-related series of diseases. In this study, we reported 10 cases of *PRRT2* gene mutations and described their clinical features, laboratory test results, treatment medications, mutated gene types, and PRRT2 and SNAP25 mRNA expression levels. Related protein structures were predicted and the mutation maps of PRRT2 reported to date were systematically summarized, which provides a reference for further research.

## Materials and methods

### Ethics approval statement

This study was approved by the Ethics Committee of the Shanghai Children's Hospital (Ethics number: 2019R071-F03), affiliated with the Shanghai Jiao Tong University School of Medicine/Shanghai Children's Hospital. All protocols followed the Chinese Biological Theory Law and the Declaration of Helsinki and its subsequent amendments.

### Compliance with ethical standards

From May 2017 to February 2022, we obtained informed consent from the recruited children and their families, who signed the informed consent form. Then, we collected venous blood samples from 10 children with suspected *PRRT2* mutations and their immediate family members, and from 12 children. In addition, venous blood samples from six boys and six girls of the same age were collected as controls. The basic information of the 10 children was collected as well as the following clinical characteristics: age of onset; seizure type; past, birth, growth and development, and family history. Other information such as return data, was also collected.

### Sample collection

Peripheral venous blood (2 ml) was collected from 10 patients with suspected *PRRT2* mutations and their family members into an anticoagulant in ethylenediaminetetraacetic acid (EDTA) tubes, and genomic DNA was extracted. Peripheral venous blood samples (2 ml) from six boys and six girls in the same age group were collected simultaneously as controls.

### Exon sequencing

We collected venous blood samples from 10 children and their relatives with suspected *PRRT2* mutations. Genomic DNA was isolated from the blood samples, sheared using Covaris Ultra, and then DNA libraries were constructed and their quality was assessed. The DNA library was sequenced using an Illumina HiSeq 2,500 platform (Illumina, Inc., CA, USA) according to the manufacturer's instructions for 150-bp paired-end reads. Next, the Burrows–Wheeler alignment tool (version 0.7.15) was used to align paired-end reads based on the University of California Santa Cruz and the National Center for Biotechnology Information (NCBI) human reference genome (hg19/GRCh37).

Common variants were interpreted and filtered using Ingenuity Variant Analysis (Qiagen Inc., Germany). The identified variants were filtered out based on frequencies (minor allele frequency < 0.05) in the Exome Aggregation Consortium (http://exac.broadinstitute.org), Exome Sequencing Project (https://esp.gs.washington.edu), and 1,000 Genomes Project (http://www.1000genomes.org) databases. The results showed that 10 patients had eight previously reported frameshift (five and three cases: c.649 dup and c.649 del, respectively) mutations and one case each of a new missense (c.518A > C), and new splice (c.879 + 112G > A) mutation.

Furthermore, the new missense and splice mutations were not included in the Human Gene Mutation Database (HGMD) and Genome Aggregation Database (gnomAD). Except for patients 1 and 2, the parents of the patients declined to undergo further testing for the suspected variant. Therefore, peripheral venous blood and the DNA samples of the other children and their immediate relatives were tested using polymerase chain reaction (PCR) and Sanger sequencing to verify the existence of the variant.

### RNA extraction and reverse transcription of cDNA

RNA was extracted from 0.25 ml of fresh blood using a total RNA extraction kit according to the manufacturer's instructions (Qiagen). Then, the concentration and purity of the total RNA were determined using a nucleic acid protein analyzer. Total RNA was used as a template and reverse transcribed using the Prime Script Strand cDNA synthesis kit/reverse transcription (RT) Master Mix, according to the manufacturer's instructions. The reaction system used consisted of the following components: template RNA, 5 µl (≤500 ng); PrimeScript RT enzyme mix, 2 µl; and RNase-free water, 3 µl. The samples were incubated at 37 °C for 15 min and then 85 °C for 5 s.

### Real-time quantitative PCR (qPCR)

Fluorescent real-time quantitative PCR (qPCR) was performed using cDNA from the blood of the enrolled children, their families, and control companions using a real-time fluorescent qPCR kit (Takara Biomedical Technology) according to the manufacturer's instructions. Specifically, we detected the expression of PRRT2 in blood using the following primers: PRRT2 forward, 5′-CCAGAAACCACAGAGACCCC-3′ and reverse, 5′-CCAGAAACCACAGAGACCCC-3′ and SNAP25 forward, 5′-ACCAGTTGGCTGATGAGTCG-3′ and reverse, 5′-GTTCGTCCACTACACGAGCA-3′. Reverse transcription (RT)-qPCR was performed using SYBR Premix Ex Taq II (Tli RNase H plus; Takara Shuzo Co., Ltd., Japan) and a LightCycler® 96 thermocycler (Roche AG, Switzerland).

The total reaction volume was 20 µl and the mixture consisted of: Heiff UNICONqPCR SYBR Green Master Mix, 10 µl, forward and reverse primers, each 0.4 µl; cDNA, 2 µl; and sterilized water, 7.2 µl. The reaction conditions for the RT-qPCR were as follows: pre-denaturation at 95 °C for 30 s, 95 °C for 30 s, 40 cycles of denaturation for 5 s, renaturation at 60 °C for 30 s, and the last extension at 65 °C for 15 s. The relative expression levels of samples were determined in triplicate and analyzed using the 2−*ΔΔ*Ct method. Results are shown as the means ± standard error of three experiments and differences were considered statistically significant at *P* ≤ 0.05.

### Frameshift mutation and missense mutation protein structure prediction using iterative threading assembly refinement (I-TASSER) server

We predicted the structures of the PRRT2 and wild-type proteins with frameshift (c.649 dup, c.649 del) and missense (c.518A > C) mutations using the Iterative Threading Assembly Refinement (I-TASSER) suite pipeline. This platform comprises four general steps: thread template identification, iterative structural assembly simulation, model selection and optimization, and structure-based functional annotation. The server was located at http://zhanglab.ccmb.med.∼umich.edu/I-TASSER/.

### Conserved sequence analysis

We analyzed the PRRT2 mutein sequences of *Homo sapiens*, *Marmota monax*, *Pan troglodytes*, *Macaca mulatta*, *Myodes glareolus*, *Myotis brandtii*, *Mustela putorius furo*, *Rattus norvegicus*, and *Mus musculus* to predict these sites of conservatism.

## Results

### Clinical data

The sex, age of onset, clinical manifestations, diagnosis, and clinical information on head magnetic resonance imaging (MRI), VEEG, treatment and outcome, *PRRT2* gene mutation sites, and parental verification are summarized in Table 1. The 10 children who were analyzed with suspected *PRRT2* mutations all had heterozygous mutations, consisting of four mutations. Two of the mutations were previously reported (c.649 dup, p.Arg217Profs*8; c.649 del, p.Arg217Glufs*12), whereas two were not (c.518A > C, p.Glu173 Ala; c.879 + 112G > A, p.?). All 10 children had neurological-related episodic diseases, including five cases of BFIE, three of PKD, and one each of focal impaired awareness seizures and infantile spasms with intellectual disability, whereas two patients (P3 and P6) had a history of convulsions in childhood.

**Table 1:** Summary of clinical data of 10 children with proline-rich transmembrane protein 2 (PRRT2)-related neurological disorders.

Case	Age of onset	Sex	Clinical manifestations	Nucleotides	Amino acid changes	Reported	Type of mutation	Father	Mother	Head MRI	VEEG	Medication	Evolution
P1	5 years	M	EP, focal impaired awareness seizures	c.518A > C	*p* Glu173 Ala	No	VUS	/	/	Delayed myelination	Normal	VPA,30 mg/kg.d → OXC,25 mg/kg.d;LEV,35 mg/kg.d	Control
P2	6 months	M	BFIE, GTCS → after relapse focal impaired awareness seizures	c.649 dup	p.Arg217Profs*8	Yes	Pathogenic	/	/	Delayed myelination	Normal	VPA,20-25 mg/kg.d(2006-2011) → OXC,20 mg/kg.d	Control
P3	11 years	F	PKD, Upper extremity shaking; 1 seizure within 1 year of age	c.649 del	p.Arg217Glufs*12	Yes	Pathogenic	None	Het	Normal	Normal	OXC,12 mg/kg.d	Control
P4	9 months	M	BFIE, GTS	c.649 dup	p.Arg217Profs*8	Yes	Pathogenic	None	Het	Normal	Normal	VPA,21 mg/kg.d	Control
P5	6 months	M	BFIE, focal impaired awareness seizures	c.649 dup	p.Arg217Profs*8	Yes	Pathogenic	None	Het (febrile seizures)	Small patchy abnormal signal in right occipital lobe	Normal	LEV,27 mg/kg.d → VPA,22 mg/kg.d	Control
P6	9 years	M	PKD, Right limb shaking, 4 febrile seizures within 1 year of age	c.649 dup	p.R217PfsTer8	Yes	Pathogenic	None	None	Small pituitary	Normal	OXC,10 mg/kg.d	Improvement (fewer attacks, less severe)
P7	8 years	M	PKD, shaking feet when standing, falling down suddenly	c.649 del	p.Arg217Glufs*12	Yes	Pathogenic	/	None	Normal	Normal	OXC,15 mg/kg.d	Control
P8	3 months	F	IS, Epileptic spasm, nodding and hugging spasm	c.879 + 112G > A	p.?	No	VUS	Het	None	Normal	Abnormal	TPM,4 mg/kg.d, ACTH,2U /kg.d, Use for 2 weeks → VPA, 30 mg/kg.d	Improvement (fewer attacks, less severe)
P9	4 months	F	BFIE, GTCS	c.649 del	p.Arg217Glufs*12	Yes	Pathogenic	Het (EP)	None	Delayed myelination	Abnormal	LEV,25 mg/kg.d → TPM,3 mg/kg.d	Control
P10	5 months	F	BFIE, GTS	c.649 dup	p.Arg217Profs*8	Yes	Pathogenic	Het (PKD)	None	Bilateral temporal extracerebral space widening	Abnormal	LEV,30 mg/kg.d → VPA,18 mg/kg.d	Control

EP, epilepsy; BFIE, benign familial infantile epilepsy; PKD, paroxysmal kinesigenic dyskinesia; IS, infantile spasms; GTCS, generalized tonic–clonic seizure; GTS, generalized tonic seizures; OXC, oxcarbazepine; VPA, valproic acid; LEV, levetiracetam; TPM, topiramate; ACTH, adrenocorticotropic hormone; Het, heterozygous.

Three patients (P8, P9, and P10) also exhibited abnormal EEGs. Four patients (P3, P4, P7 and P8) had normal head MRI, and the remaining four patients also experienced abnormalities of bilateral lateral ventricle retrohorn signals (P1, P2, P6 and P9), small patchy abnormal signals in the right occipital lobe (P5), and bilateral temporal extracerebral space widening (P10). The age at first onset of disease ranged from 3 months to 11 years, with an average age of 3 years and 6 months. BFIE seizure types were generalized tonic–clonic, generalized tonic, and focal impaired awareness seizures. PKD episodes were characterized by limb tremors, sudden falls, and dance-like movements of the upper limbs. Ten children received medications such as valproic acid, oxcarbazepine, levetiracetam, topiramate, and adrenocorticotropic hormone (ACTH).

In addition to two patients who relapsed 3 years after drug withdrawal, patients 6 and 8 showed reduced seizure frequency and severity, whereas the remaining patients had controllable conditions and no clinical episodes. All the patients had a normal birth history. Patient 8 exhibited delayed growth and development and at 4 months old, while she could support her head, she did not chase objects and would not laugh. At the time of this publication, the child was 14 months old and still could not sit unaided, turn over, crawl, babble, or take initiative, such as grabbing objects. The other children had a normal growth and developmental histories (supported their heads at 3 months, sat alone at 6 months, and walked at 12 months of age). Six patients(P1, P2, P3, P4, P6, and P7) had normal education levels, and three patients (P5, P9, and P10) had not yet entered school due to their young age. Patient 8 required ongoing special care and rehabilitation training. Among the 15 immediate family members, six *PRRT2* gene carriers were identified, and three had a history of febrile convulsions, seizures, and PKD. The remaining 3 members had normal clinical phenotypes. Next, 4 cases were selected to introduce 4 different locus mutations.

Case 1: A novel C.518A > C (p.Glu173 Ala) PRRT2 mutation

He was a boy who was born to a gravida 1 para 1 (G1P1) mother at full term and had a birth weight of 3,950 g. His past, growth and development, and family history were normal. Onset of disease occurrence was at the age of 5 years, with focal impaired awareness seizures as the main manifestation, which occurred during sleep. The specific manifestations are binocular strabismus, deviation of the corners of the mouth, upper limb stiffness and shaking, lasting about 1–2 min to relieve themselves. VEEG showed that the slow activity of the anterior head increased and the waking period was obvious. The brain MRI showed bilateral lateral ventricle posterior horn films and delayed myelination could be considered if the abnormal signal was abnormal. Genetic testing suggested that the patient carried a novel c.518A > C (p.Glu173 Ala) PRRT2 mutation, which has not been previously reported in the literature. Initially, antiepileptic treatment entailed valproic acid, but the seizure was not controlled. After antiepileptic treatment with oxcarbazepine combined with levetiracetam, the seizure did not reoccur, and the VEEG was normal.

Case 2: A c.649 dup (p.Arg217Profs*8) PRRT2 mutation

Case 2 was a boy with normal birth history and a disease onset age of 6 months. He carried a *PRRT2* mutation, c.649 dup (p.Arg217Profs*8), which has been reported previously (1, 2, 16). This patient began to experience convulsive seizures at 6 months of age, which manifested as generalized tonic-clonic seizures. Specific manifestations were eyes staring, mouth closed, loss of consciousness, hands shaking, lasting about 1 min to relieve themselves. The VEEG showed no abnormalities and an MRI of the head showed abnormal signals in the posterior horns of the bilateral ventricles, suggesting the possibility of delayed myelination. The patient did not have any seizures after antiepileptic treatment with VPA, and the drug was discontinued after 5 years of regular treatment. However, 3 years after drug withdrawal the convulsions reappeared and manifested as focal impaired awareness seizures. A review of the patient's VEEG showed no abnormalities and the head MRI was normal.

Case 3: A c.649 del (p.Arg217Glufs*12) PRRT2 mutation

This patient was a girl with a normal birth history, but who had a history of convulsions within 1 year of her birth. She was admitted to the hospital because of repeated episodes of upper limb shaking when changing her body position, similar to dance-like movements, occasionally accompanied by limb stiffness and falling while walking. The age of disease onset was 11 years, and the patient carried a c.649 del (p.Arg217Glufs*12) *PRRT2* gene mutation, which was previously reported by van Strien et al. (17–19). VEEG and head MRI showed no abnormalities. The patients symptoms were relieved after the administration of oxcarbazepine and the seizures stopped.

Case 8: A novel c.879 + 112G > A(p.?) PRRT2 mutation

Case 8 was a full-term baby girl delivered by cesarean section in puerpera G2P2. There was no rescue or asphyxia during delivery, and no febrile convulsions were repeated 3 months after delivery. Performance into a generalized epileptic spasm, sudden series of nodding and hugging spasms, the number of strings varied daily, 10–20 for each string. The child also exhibited developmental delays, where she raised her head at 4 months, did not chase objects, and did not laugh. At 14 months old, the child still could not sit unaided, turn over, crawl, babble, or take the initiative to grab objects. The VEEG prompts showed hypsarrhythmia index of 70%–80% in each phase, indicating the presence of electrical status epilepticus in sleep (ESES) during which four isolated spastic seizures were observed during the awake period. The brain MRI was normal. Genetic testing revealed that the child carried a novel c.879 + 112G > A(p.?) *PRRT2* mutation that has not yet been reported in the literature. Parental testing results showed that the mutation was inherited from the father who had a normal clinical phenotype. After treatment with topiramate and ACTH, the clustered seizures turned into single seizures occurring 8–10 times/day. After 1 month, VPA was added to the combination and the number of attacks gradually reduced to 2–3 times/day.

### qPCR expression analysis of *PRRT2* and *SNAP25* genes

**Figure 1:**
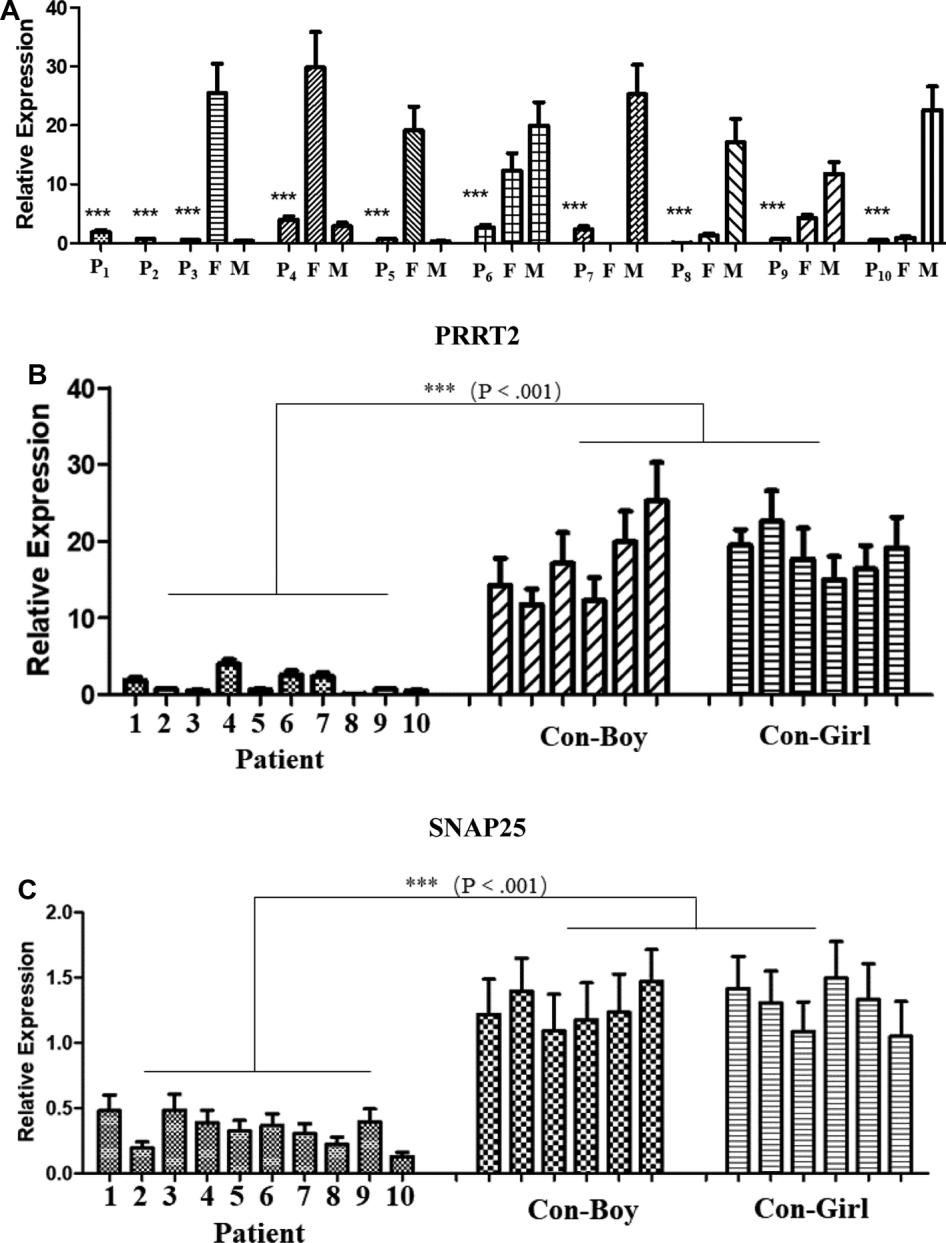
Quantitative polymerase chain reaction (qPCR) analysis of proline-rich transmembrane protein 2 (*PRRT2*) and synaptosome-associated protein 25 (*SNAP25*) gene expression. P1–P10, patients 1–10; F, father; M, mother; CON-Boy, control boys; CON-Girl, control girls. (***P ≤ 0.001). *PRRT2* expression level in (**A**) patients and their parents and (**B**) patients and control children of the same age. (**C**) *SNAP25* expression level in patients and control children of the same age.

The expression level of PRRT2 in the children and carriers was significantly lower than that in individuals without mutations (Figure 1A). The expression levels of the *PRRT2* and *SNAP25* genes in the 10 children were significantly lower (*P* ≤ 0.001) than those in the control group of the same age were (Figure 1B,C). Further in-depth analysis showed no significant difference in the expression of *PRRT2* and *SNAP25* between the two groups with the c. 649 dup and c. 649 del mutations (*P* > 0.05). The clinical phenotype analysis showed no difference in the expression of *PRRT2* and *SNAP25* between the BFIE and PKD disease groups (*P* > 0.05).

### Protein structural changes and conservation analysis

**Figure 2:**
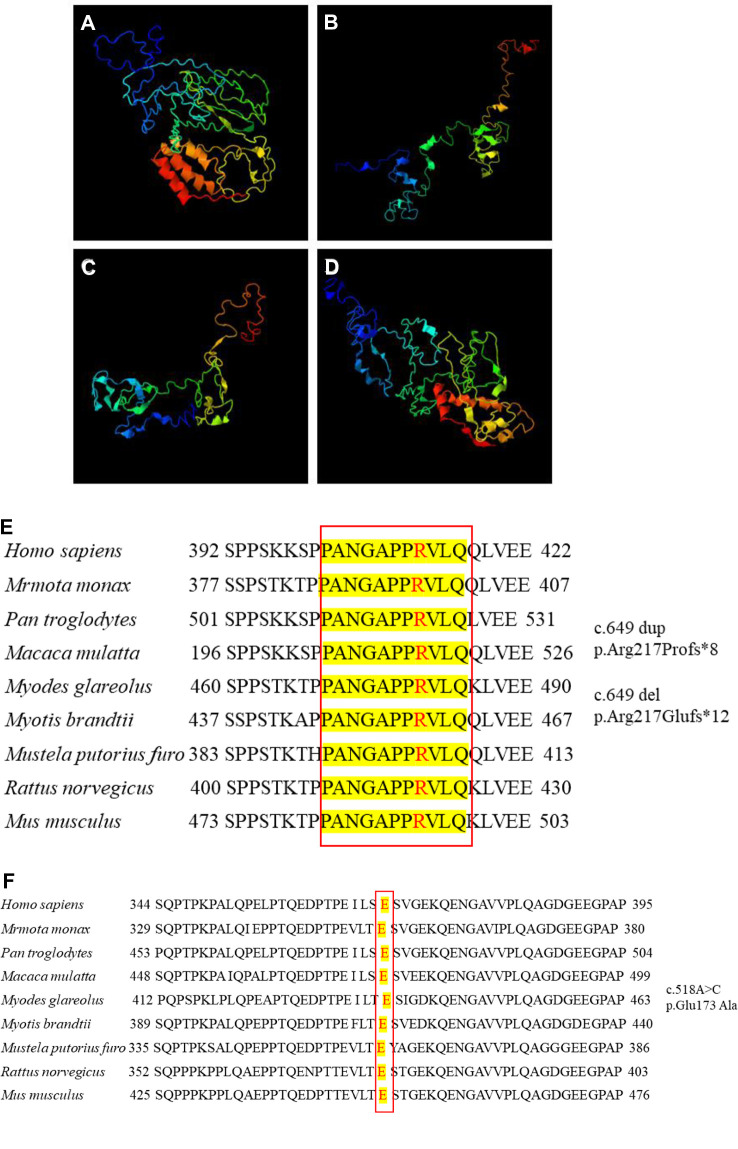
Three-dimensional structure of proline-rich transmembrane protein 2 (PRRT2). (**A**) Wild-type protein. Frameshift mutation (**B**) c.649 dup and (**C**) c.649 del. (**D**) Missense mutation c.518A > C. Conservation studies of (**E**) c.649 and (**F**) c.518 on *Homo sapiens*, *Marmota monax*, *Pan troglodytes*, *Macaca mulatta*, *Myodes glareolus*, *Myotis brandtii*, *Mustela putorius furo*, *Rattus norvegicus*, and *Mus musculus*.

We further compared the three-dimensional structure of PRRT2 proteins with those of the wild-type (Figure 2A) and frameshift 649 dup (Figure 2B), 649del (Figure 2C), and missense (Figure 2D) mutations at http://zhanglab.ccmb.med. The frameshift mutation of the duplication and deletion at position 649 caused premature translation termination, truncation of the protein sequence, and significant structural changes. The missense mutation c.518 A > C resulted in the conversion of the amino acid glutamic acid (Glu) to alanine (Ala), which was located in the proline (Pro)-rich domain. Furthermore, the three-dimensional structure of the mutant was different from that of the wild-type. In this study, *PRRT2* mutation sites were conserved in *H. sapiens*, *M. monax*, *P. troglodytes*, *M. mulatta*, *M. glareolus*, *M. brandtii*, *M. putorius furo*, *R. norvegicus*, and *M. musculus* (Figures 2E,F). The results showed that the c.649 and c.518 mutations were relatively stable.

### PRRT2 domain and mutation map

**Figure 3:**
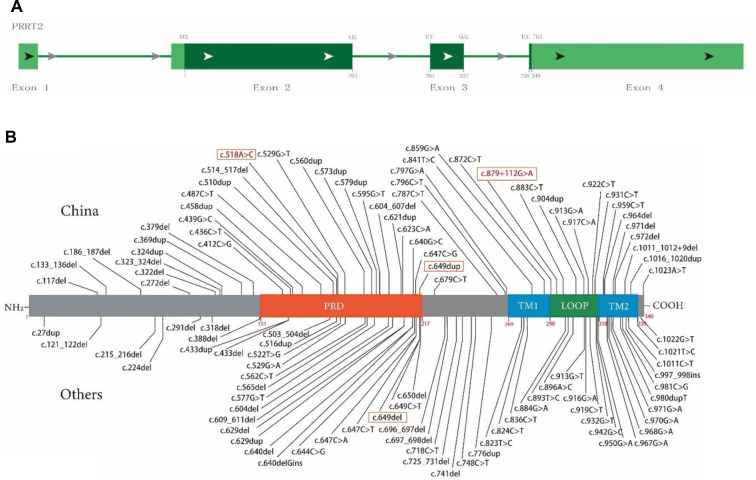
Schematic diagram of proline-rich transmembrane protein 2 (PRRT2) mutation map and protein domain superposition. (**A**) PRRT2 is composed of four exons, exons 2–4 encode multidomain protein containing 340 amino acids. (**B**) Main domains of PRRT2 protein: PRD (orange, 131–216), proline-rich domain; TM1 (blue, 269–289), transmembrane domain 1; TM2 (blue, 318–338), transmembrane domain 2; Loop (green, 290–317), domain between TM1 and TM2. Text box shows mutations reported in this article, where red font indicates new mutation.

PRRT2 consists of four exons, and exons 2–4 encode a multidomain protein containing 340 amino acids (Figure 3A). The N-terminal extracellular domain (amino acids 1–268) contains a Pro-rich domain (PRD; amino acids 131–216), and the C-terminal domain has two transmembrane domains (amino acids 268–289 and 318–338, Figure 3B).

To date, 105 PRRT2 variants have been included in the HGMD (April 2022, Figure 3A), including 52 frameshift mutations, accounting for 49.5% (with 19 and 33 insert and delete frameshifts, respectively), distributed throughout the sequence. These also include 53 point mutations, accounting for 50.5% (with 38 and 15 missense and nonsense mutations, respectively), and the distribution trend is concentrated in the C-terminus. Figure 3B illustrates the structure and the upper part was first reported in China and the lower part was first reported in other countries.

## Discussion

In this study, we investigated the clinical features and role of the underlying *PRRT2* genetic mutations in 10 children with related neurological diseases. Among them, 5 and 3 had BFIE and PKD, respectively, whereas 1 each had focal impaired awareness seizures with epilepsy and infantile spasms with intellectual disability. Three children had a positive family history and their parents had experienced episodic abnormalities, febrile convulsions, and epilepsy when they were young. The mutation site c.649 dup was identified in five patients, c.649 del in three, and the other two had new mutations at sites c.518 and c.879 + 112. The PRRT2 mutation map was also updated further. The analysis of the three-dimensional structure of the mutant in this study indicated it differed from that of the wild-type and, therefore, we speculated that these changes may affect the intrinsic stability of proteins and their protein–protein interactions.

*PRRT2* has been identified as a causative gene of paroxysmal neurological genetic spectrum disorders (1, 3), mainly in the cerebral cortex, basal ganglia, hippocampus, and cerebellum (20). The most common clinical phenotypes are BFIE, PKD, and ICCA, which are autosomal dominant, with incomplete penetrance. In addition, approximately 5% of patients develop other phenotypes, including various paroxysmal dyskinesias, infantile seizures, paroxysmal torticollis, migraine, hemiplegic migraine, and episodic ataxia (12). Ebrahimi-Fakhari et al. (21) performed a pooled analysis of 1,444 children with 70 different *PRRT2* mutations and found that the most common mutation site was c.649dup, followed by c.649del. Furthermore, they reported that 5% of the cases were *de novo*, whereas 87.1% were unified by either parent (21). In addition, 94.7% of the diagnoses in that study were consistent with the BFIE-PKD/IC-PKD spectrum, whereas 80% and 49.5% of children with PKD and BFIE, respectively had a positive genetic family history. In addition, Danique RM Vlaskamp et al.(22) found that a 16p11.2 deletion including PRRT2 or PRRT2 loss-of-function sequence variant has the same underlying loss-of-function disease mechanism.

We discovered that 105 *PRRT2* variants have been reported to date through a search of the HGMD human gene mutation database (2,022.4), which included 52 (49.5%) frameshift mutations and 53 (50.5%) point mutations. Although frameshift mutations are distributed throughout *PRRT2*, missense mutations are mainly concentrated at the C-terminus. Given the order of distribution of the main domains in *PRRT2*, localization of the truncation to the plant homeodomain (PHD) also affects the changes in the transmembrane domain.

Therefore, the number of missense mutations in a particular region is indicative of the functional importance of that region, and 22 frameshift and 18 missense mutations were found in the PHD. Six frameshift and 16 missense mutations were found in transmembrane domain 1 and 2 (TM1 and TM2), respectively, suggesting that the latter may play a more important role in the biological function of PRRT2. Zhao et al. (23) studied the function and pathogenicity of PRRT2 missense variants in related diseases. Furthermore, 15 of the 29 *PRRT2* missense variants were classified as likely pathogenic and were concentrated in the C-terminus, suggesting that the PRRT2 C-terminus is very important for its physiological function.

The pathogenic mechanisms of PRRT2-related diseases remain unclear. Previous evidence indicates that PRRT2 interacts with SNAP25 at the presynaptic membrane and may be involved in synaptic vesicle fusion and neurotransmitter release (3, 20). As a functional domain of the target membrane-soluble N-ethylmaleimide attachment protein receptor (t-SNARE) protein, SNAP25 is involved in intrasynaptic vesicle fusion and regulates the voltage-gated Ca^2+^ channels. In addition, in nerve endings, *PRRT2* gene mutations also affect Na^+^ and Ca^2+^ channels on the cell membrane, resulting in abnormal cell membrane potential. In subcellular divisions of the mouse brain, PRRT2 was found to be co-localized with SNAP25 and presynaptic region-associated proteins (7).

Corradini et al. (24) found that a reduced expression of SNAP25 in transgenic heterozygous mice was associated with hyperkinesia and increased abnormal EEG discharge. Therefore, in this study, we measured the expression levels of PRRT2 and SNAP25, and found that their expression levels were significantly lower in patients than in the control group. PRRT2 has been suggested to interact with SNAP25, causing a significant decrease in its expression, which may cause protein truncation or activate nonsense-mediated mRNA degradation. These effects lead to a loss of function or abnormal localization of protein products, resulting in the occurrence of diseases (25).

Patients carrying *PRRT2* gene mutations respond well to drug treatment and with both BFIE and PKD, most patients can completely control their seizures with single-drug antiepileptic drug treatment. In this study, the first choice of levetiracetam (LEV) treatment in the three children with BFIE failed to control the seizures, which even increased after treatment; however, the seizures were quickly controlled after switching the treatment to VPA or topiramate (TPM). This may be related to the pathogenic mechanism of PRRT2 and the mechanism of action of LEV. Studies evidence demonstrates that LEV downregulates the release of intracellular Ca^2+^ caused by glutamate and upregulates Na^+^/K^+^ ATPase (26).

This may be the reason for the poor effect of LEV treatment or even the aggravation of episodes. Therefore, ion channel blockers, such as oxcarbazepine (OXC) and VPA, are the first choice for the treatment of children with episodic diseases caused by *PRRT2* gene mutations (15). The advantages of ion channel blockers are that their adverse reactions are significantly lower than those of the other agents and they have no significant effect on cognitive function (15). In this study, the VEEG did not show evidence of extensive abnormal discharge, and OXC was used as the drug of choice for controlling episodic symptoms, which is consistent with the results of previous studies (27).

In conclusion, the *PRRT2* gene has considerable diversity that can lead to a series of diseases, and a clear relationship between genotype and clinical phenotype has not yet been established. Future further studies of the *PRRT2* mutation in a larger number of patients with related diseases would provide a clearer and more accurate correlation. This information could be used to predict the clinical phenotype of carriers, guide prenatal and postnatal care, and provide a reference value for disease treatment and prognosis. Finally, further study of the pathogenic mechanism of PRRT2 would provide a reference for precision treatment.

## Data Availability

The raw data supporting the conclusions of this article will be made available by the authors, without undue reservation.
